# Dataset of volatile compounds in fresh and stored cut watermelon (*Citrullus lanatus*) under varying processing and packaging conditions

**DOI:** 10.1016/j.dib.2019.104299

**Published:** 2019-08-22

**Authors:** Michelle Louise Mendoza-Enano, Roger Stanley, Damian Frank

**Affiliations:** a Australian Research Council Training Centre for Innovative Horticultural Products, Life Science Bldg, College Rd., University of Tasmania, Hobart, 7005, Tasmania, Australia; b Centre for Food Innovation, Newnham Campus, University of Tasmania, 7250, Launceston, Tasmania, Australia; c Commonwealth Scientific and Industrial Research Organisation, 11 Julius Ave., North Ryde, 2113, New South Wales, Australia

**Keywords:** Fresh-cut, Lidding film, MAP, Post-cut sanitation, PTR-MS, SPME GC-MS, Watermelon

## Abstract

Headspace volatile data for fresh and stored cut watermelon measured by solid phase microextraction gas chromatography - mass spectrometry (SPME GC-MS) and also proton transfer reaction-mass spectrometry (PTR-MS) are reported [1]. Eight different processing and packaging storage treatments were applied to fresh and stored cut watermelon including varying the processing treatments (with vs. without post-cut sanitation spray), headspace gas composition (ambient vs. modified atmosphere), lidding film permeability (perforated vs. non-perforated), storage temperature (3 and 7 °C) for up to 8 days. A total of 41 volatile compounds were characterized by SPME GC-MS in watermelon headspace on the basis of their electron impact (EI) mass spectra. Reference chemical standards and matching linear retention indices (LRIs) were used to confirm the identity of 32 volatiles (Supplementary Table 1). PTR-MS fragmentation data for 32 key odor-active reference volatiles identified in watermelon are reported (Supplementary Table 2). PTR-MS fragment data for fresh and stored cut watermelon are provided (Supplementary Table 3).

**Table:** Specifications Table

Subject	*Agricultural, Food science*
Specific subject area	*Food chemistry, Flavor chemistry*
Type of data	*Tables in Microsoft Excel Worksheet format*
How data were acquired	*Volatile compounds in fresh and stored cut watermelon were concentrated using headspace solid phase microextraction and analyzed by gas chromatography- mass spectrometry (Shimadzu QP-2010 Plus GC/MS, Tokyo, Japan) and by high sensitivity single quadrupole proton transfer reaction – mass spectrometry (PTR-MS, Ionicon Analytik GmbH, Innsbruck, Austria).*
Data format	*Analyzed, semi-quantitative data*
Parameters for data collection	*Fresh-cut red seedless watermelon (Citrullus lanatus* cv. Royal Armada*) was packed in different packaging formats and stored under different temperatures and time periods.*
Description of data collection	*Volatile compounds (n = 41) were measured in watermelon headspace by solid phase microextraction (SPME) and electron impact gas chromatography-mass spectrometry (GC-MS)* *32 volatiles were confirmed by matching reference volatile linear retention indices (LRIs)* *Semi-quantitative peak area data (ng g*^*−1*^*) across different storage treatments* *Mass fragmentation patterns of 32 reference volatile compounds using PTR-MS* *Semi-quantitative PTR-MS fragment data for different storage treatments*
Data source location	*Commonwealth Scientific and Industrial Research Organisation* *North Ryde, NSW, Australia*
Data accessibility	Data are provided as supplementary Excel tables within the article.
Related research article	Author's name: Mendoza-Enano, M. L., Stanley, R. and Frank, D. Title: Linking consumer sensory acceptability to volatile composition for improved shelf-life: A case study of fresh-cut watermelon (Citrullus lanatus) Journal: Postharvest Biology and Technology DOI: https://doi.org/10.1016/j.postharvbio.2019.03.018

**Table:** 

**Value of the Data** • The SPME GC-MS data provide reference volatile profiles for freshly cut watermelon and after storage under different conditions. • PTR-MS reference fragmentation data provided for 32 watermelon volatiles is a valuable reference for other researchers in postharvest biology and technology. • The data will be useful for the planning of other postharvest interventions to maintain watermelon (and other fresh-cut fruit) quality.

## 1 Data

The Microsoft Excel Worksheet provided as supplementary data for this article include the following data sets: Mean (n = 3) semi-quantitative SPME GC-MS volatile composition data (ng g^−1^) of fresh and stored cut watermelon samples and p-value for comparison of various processing and packaging treatments (Supplementary data, Table 1), mass fragmentation patterns of 32 chemical reference volatile compounds obtained using PTR-MS (Supplementary data, Table 2), and normalized mean (n = 2) of PTR-MS data for fresh-cut watermelon samples at different processing, packaging and storage conditions (Supplementary data, Table 3).

## 2 Experimental design, materials, and methods

### 2.1 Design

Eight different processing and packaging storage treatments were applied to fresh and stored cut watermelon including varying the processing treatments (with vs. without post-cut sanitation spray), headspace gas composition (ambient vs. modified atmosphere), lidding film permeability (perforated vs. non-perforated), storage temperature (3 and 7 °C) for 1, 6 and 8 d, including fresh sample (0 d) as experimental control [1]. Three replicate samples for each treatment stored at 3 °C for 8 d were used for headspace SPME GC-MS analysis. A total of 86 unopened watermelon cup samples (n = 2) were tested for the headspace volatile profiling using PTR-MS.

The following treatment codes were used for SPME data analysis:

Fresh = without post-cut sanitation, packed in ambient air and perforated film (polyester-based multi-laminated film with 6 × 150 μm diameter perforations per cup);
AnT = without post-cut sanitation spray, packed in ambient air and non-perforated film (polyester-based multi-laminated film without perforation);
AN = with post-cut sanitation spray, packed in ambient air and non-perforated film polyester-based multi-laminated film without perforation):
AP = with post-cut sanitation spray, packed in ambient air and perforated film (polyester-based multi-laminated film with 6 × 150 μm diameter perforations per cup);
MP = with post-cut sanitation spray, packed in modified atmosphere and perforated film (polyester-based multi-laminated film with 6 × 150 μm diameter perforations per cup);
Ap = with post-cut sanitation spray, packed in ambient air and perforated film (polyester-based multi-laminated film with 6 × 75 μm diameter perforations per cup);
Mp = with post-cut sanitation spray, packed in modified atmosphere and perforated film (polyester-based multi-laminated film with 6 × 75 μm diameter perforations per cup); and
MN = with post-cut sanitation spray, packed in modified atmosphere and non-perforated film (polyester-based multi-laminated film without perforation).

An example of sample naming for PTR-MS data is shown:

AnT-3-1 = without post-cut sanitation spray, stored at 3 °C for 1 d.

## 3 Materials

Fresh-cut red seedless watermelons (cv. Royal Armada) were either subjected to post-cut sanitation spray (150–200 mg L^−1^ peracetic acid) or none, packed with either ambient air or modified atmosphere (5 %O_2_, 10 %CO_2_ and 85 %N_2_) in polyethylene plastic cups, sealed with either perforated (6 × 150 μm diameter perforations or 6 × 75 μm diameter perforations) or non-perforated lidding film and stored at either 3 or 7 °C and sampled at 1, 6 and 8 d.

The following reference chemicals (≥98 %purity) were supplied by Sigma Aldrich (Castle Hill, NSW, Australia): hexanal, 1-heptanol, *(E)*-2-hexenal, octanal, dimethyl trisulfide, 2-pentylfuran, *(E,Z)*-2,6-nonadienal, 1-octen-3-one, 1-octen-3-ol, *(Z)*-2-penten-1-ol, *(E,E)*-2,4-heptadienal, *(Z)*-6-nonen-1-ol, 3-octanone, *(Z)-*6-nonenal, 1-nonanol, 2-butanone, 3-methylbutanal, ethanol, *(**d**)*-limonene, 3-methylbutanol, *(E)*-2-heptenal, 1-hexanol, *(Z)*-3-hexen-1-ol, nonanal, acetic acid, decanal, *(β)*-linalool, acetophenone, and 4-methyl-1-pentanol (internal standard, IS). Other volatile compound references such as 1-penten-3-ol, *(E)*-2-octenal, *(E)*-2-nonenal and 1-penten-3-one were obtained from Givaudan (ex-Quest International). Helium gas and compressed air were supplied by Coregas (Sydney, Australia). Silane treated glass wool and saturated alkanes standard C7–C40 came from Supelco (Bellefonte, USA). Tenax® porous polymer adsorbent (Tenax-TA, 60/80 mesh) were provided by Sigma-Aldrich (Castle Hill, Australia) and Milli-Q water was obtained from Synergy UV Millipore (Sydney, Australia).

## 4 Sample preparation

### 4.1 Samples for SPME-GC/MS analysis

Watermelon cubes (2.5 cm × 2.5 cm x 2.5 cm) from unopened individual cups (n = 2 replicates) stored at 3 °C for 8 d and previously used for PTR-MS analysis were macerated using a handheld food processor and stored in 50 mL disposable polypropylene tubes (Rowe Scientific, NSW, Australia). These samples were frozen at −80 °C for up to 30 d for SPME-GC-MS analysis. Samples were thawed for 30 min at 25 °C in a water bath on the day of analysis. Two grams of each sample were placed in 20 mL headspace vial, added with 10 μL 4-methyl-1-pentanol, 40 μg mL^−1^ internal standard (IS) and immediately sealed with a gas-tight Teflon lined septum.

### 4.2 Samples for PTR-MS analysis

Unopened individual watermelon cups (n = 2 replicates) representing various processing, packaging and storage treatments listed in section 2.1 were directly scanned by the PTR-MS. Reference volatile standards (when available) were also scanned to determine the PTR-MS ion fragments of the main odor-active compounds of watermelon. For each reference standard, 1 μL was added to 10 mL Milli-Q water in 250 mL Schott bottle.

## 5 Volatile compound analysis

### 5.1 Headspace SPME GC-MS analysis

Headspace analysis was performed with an auto-sampler AOC-5000 Shimadzu (Tokyo, Japan), using Divynylbenzene/Carboxen/Polydimethylsiloxane SPME fibers (23-gauge, 2 cm, Agilent Technologies, Bellefonte, USA). Volatiles were extracted from each treatment (n = 3 samples) prepared in a 20 mL headspace vial and incubated at 40 °C for 20 min. The SPME fiber was injected and desorbed in a splitless mode at 250 °C for 5 min (initially held at 35 °C for 5 min) onto the SPME GC-MS (Shimadzu QP-2010 Plus, Tokyo, Japan). Zebron-WAX column (30 m, 0.25 mm and 0.50 μm; Phenomenex, Lane Cove West, Australia) was used for the separation of compounds and volatile detection was performed under EI mode at 70 eV over the mass range *(m/z)* 40–250. The volatile compounds were identified based on their retention times in gas chromatogram and peaks were matched with EI mass spectral in the National Institute of Standards and Technology (NIST) 14 mass spectral library using NIST mass spectral search program version 2.0 (United States of America, 2002) and confirmed by the linear retention indices (RI) of reference compounds. Integrated area peak of each compound was normalized to the IS and semi-quantitative data (ng g^−1^) were estimated prior to statistical analysis. Previous published work [2], [3], [4], [5], [6], [7], [8], [9], [10], [11], [12], [13] on the volatile profiles of watermelon were considered in evaluating the GC-MS data in this study (see Supplementary multimedia component 1).

### 5.2 PTR-MS analysis

Volatile profile was assessed by a high sensitivity single quadrupole PTR-MS (Ionicon Analytik GmbH, Innsbruck, Austria). Mass spectra was recorded in the mass-to-charge *(m/z)* range of 35–200 with a dwell time of 100 ms per *m/z*, and a cycle time of 120 s. The drift tube was held at 600 V, 2.21 mbar, and 70 °C.

For each unopened watermelon cup sample, two stainless steel needles were inserted into the lidding film of each cup: 0.45 × 13 mm needle (26 G x 1.5”; Terumo Corporation, NSW, Australia) to allow flow through of gases and 0.70 × 38 mm needle (22 G x 1.5″; Terumo Corporation, NSW, Australia) attached to the PTR-MS inlet PEEK tubing using a Teflon® Luer-lock fittings to sample headspace volatiles. For chemical reference standards, Schott bottle was connected to a PTR-MS inlet tubing (PEEK – 0.25 mm id, Upchurch Scientific, USA). Other inlets were connected via Teflon® Luer-lock fittings. The measurement proceeded with an air flow of 100 mL min^−1^ for 20 cycles under the conditions described above. Some reference PTR-MS ions were obtained from literature [14].
